# Strategies for the Identification and Tracking of *Cronobacter* Species: An Opportunistic Pathogen of Concern to Neonatal Health

**DOI:** 10.3389/fped.2015.00038

**Published:** 2015-05-05

**Authors:** Qiongqiong Yan, Séamus Fanning

**Affiliations:** ^1^UCD-Centre for Food Safety, WHO Collaborating Centre for Research, Reference and Training on Cronobacter, School of Public Health, Physiotherapy and Population Science, University College Dublin, Dublin, Ireland

**Keywords:** *Cronobacter* species, molecular identification, whole genome sequencing, DNA microarray, high-throughput whole-transcriptome sequencing

## Abstract

*Cronobacter* species are emerging opportunistic food-borne pathogens, which consists of seven species, including *C. sakazakii*, *C. malonaticus*, *C. muytjensii*, *C. turicensis*, *C. dublinensis*, *C. universalis*, and *C. condimenti*. The organism can cause severe clinical infections, including necrotizing enterocolitis, septicemia, and meningitis, predominately among neonates <4 weeks of age. *Cronobacter* species can be isolated from various foods and their surrounding environments; however, powdered infant formula (PIF) is the most frequently implicated food source linked with *Cronobacter* infection. This review aims to provide a summary of laboratory-based strategies that can be used to identify and trace *Cronobacter* species. The identification of *Cronobacter* species using conventional culture method and immuno-based detection protocols were first presented. The molecular detection and identification at genus-, and species-level along with molecular-based serogroup approaches are also described, followed by the molecular sub-typing methods, in particular pulsed-field gel electrophoresis and multi-locus sequence typing. Next generation sequence approaches, including whole genome sequencing, DNA microarray, and high-throughput whole-transcriptome sequencing, are also highlighted. Appropriate application of these strategies would contribute to reduce the risk of *Cronobacter* contamination in PIF and production environments, thereby improving food safety and protecting public health.

## Introduction

*Cronobacter* species (formerly known as *Enterobacter sakazakii*) are opportunistic pathogens of the family Enterobacteriaceae, which have been documented in life-threatening infections predominantly in neonates of <4 weeks of age (1). The mortality rate of *Cronobacter* infections ranges from 40 to 80%, and includes the clinical syndromes of necrotizing enterocolitis (NEC), bacteremia, and meningitis (2, 3). This bacterium has been isolated from a range of food sources including dairy-based products, dried foods such as herbal tea, flour, nuts, adult and infant cereals, herbs and spices, dried meats, rice, and others (4–6). Additionally, *Cronobacter* species have also been cultured from a variety of different sources and environments based on surveillance studies, including from common house flies, households, livestock facilities, and food manufacturing operations, in particular powdered infant formula (PIF) production facilities (7–10). Contaminated PIF has been epidemiologically linked with many of the infections reported (1, 2). The capacity to detect and identify *Cronobacter* species, differentiating them from other members of the Enterobacteriaceae, would contribute positively toward a reduction in the health risks to vulnerable individuals. This review summarizes the laboratory-based approaches that can be used to detect, and trace this pathogen of importance to neonatal health.

## Conventional Culture Strategies

The conventional culture method for the identification of *Cronobacter* species was first reported by Muytjens et al. (11). The International Organization for Standardization (ISO) and International Dairy Federation (IDF) published a technical standard protocol, known as ISO/TS 22964, for the detection of *Cronobacter* species from milk-based powdered formula.^1^ The US Food and Drug Administration (US-FDA) later established both a culture method for the detection/isolation of *Cronobacter* spp. and a real-time PCR method for rapid screening (12). The procedures and flowchart for the sample preparation, isolation, confirmation, and identification of *Cronobacter* species have been described in detail.^2^ Both ISO/TS 22964 and US-FDA method are currently accepted as reference methods for the identification of *Cronobacter* species.

## Immuno-Based Detection Protocols

Efforts on immuno-based protocols have been made both commercially and within the laboratories. The enzyme-linked immunosorbent assay (ELISA) technology has been applied in the VITEK immuno diagnostic assay system (known as VIDAS, bioMérieux, France) for the detection of several organisms using kits, including *Salmonella*, *Escherichia coli* O157:H7, *Listeria* species, *Campylobacter jejuni*, and *Staphylococcus* species enterotoxins. The kits for *Cronobacter* species have been developed and early stage tests showed promising results (13).

Meanwhile, several antibody targets, such as IgG, IgY, and outer membrane protein A (OmpA), were used for the immuno detection of *Cronobacter* species (14–17). Hochel and Škvor (14) developed an indirect competitive enzyme immunoassay for the detection of *Cronobacter* species using polyclonal antibodies. The surface antigenic determinants in *Cronobacter* species using monoclonal antibodies (MAbs) and MALDI-TOF Mass spectrometry were also investigated (15). A sandwich ELISA was undertaken to detect *C. muytjensii* in PIF (16). Recently, two rapid analytical methods, including a pAb-based indirect ELISA and a sandwich ELISA using pAb and mAb, were established for the detection of *Cronobacter* species in PIF (18).

## Molecular Detection and Identification

**Figure 1:**
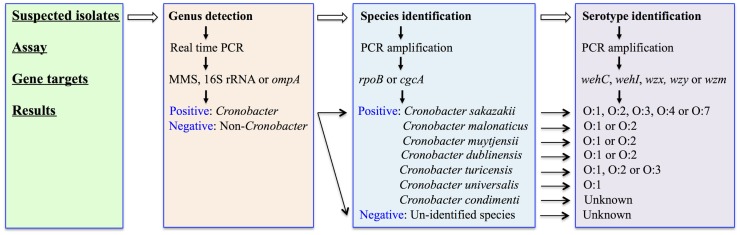
**A flowchart describes the molecular detection and identification of *Cronobacter* species at genus, species, and serotype levels**.

Molecular detection techniques have always been regarded as useful tools to extend our understanding of the epidemiology of a bacterium of importance to human health. These protocols are usually designed to target unique genes contained on the genome of the pathogen of interest. For *Cronobacter* species, the molecular detection and identification targets are designed at various levels, including the genus-, species-, and serotype-levels, respectively as described in Figure 1.

### Genus detection

*Cronobacter* species were originally known as yellow-pigmented *Enterobacter cloacae*, being reclassified subsequently as *Enterobacter sakazakii* based on deoxyribonucleic acid (DNA)–DNA hybridization, biochemical reactions, pigment production, and antibiotic susceptibility (19). The phylogenetic relationships of *Cronobacter* species to that of other members of the Enterobacteriaceae were investigated for 126 isolates using partial 16S ribosomal DNA (rDNA) and *hsp60* sequencing. These data identified four clusters within the genus and indicated substantial taxonomic heterogeneity (20). Further investigation using amplified fragment length polymorphism (f-AFLP) analysis, full-length 16S rDNA gene sequencing, DNA–DNA hybridization, in combination with phenotypic profiling, provided the grounds to consider the re-designation of *E. sakazakii* as a new bacterial genus. This was designated as *Cronobacter* species (21, 22).

A real-time PCR protocol, using a TaqMan-based design, was subsequently developed to aid in the detection of this genus. This method was originally described by Seo and Brackett (23). This approach focused on part of the macromolecular synthesis (MMS) operon (including the *dnaG* gene at 5′ end and the *rpsU* gene at 3′-end) to amplify a target sequence of 78 bp. Other strategies targeted different genes within the bacterium including 16S rRNA, *ompA*, and others (23–29). The 16S rRNA gene was later selected in a number of studies for the rapid detection of *Cronobacter* species using TaqMan (24, 27). Recently, the *ompA* gene has also been used as a target for the specific detection and rapid identification of *Cronobacter* species in PIF (28, 29). Both TaqMan and SYBR green assays were reported to be highly specific, sensitive, and efficient methods for the detection of *Cronobacter* species in infant formula-based matrices using suitable primers (25, 26).

Several commercial real-time PCR based protocols were made available, and which include the BAX^®^System PCR Assay *E. sakazakii* (DuPont, Qualicon, Wilmington, DE, USA), the Assurance GDSTM *E. sakazakii* (BioControl, Bellvue, CO, USA), and the foodproof^®^ *E. sakazakii* Detection Kit (Biotecon Diagnostics, Potsdam, Germany) (30). In comparisons with the conventional ISO and US-FDA methods, these rapid detection systems reduce the time to detect *Cronobacter* species and therefore would facilitate a positive release strategy for finish products.

### Species identification

Originally six *Cronobacter* species were defined on the basis of f-AFLP fingerprints, ribotype patterns, full-length 16S rRNA gene sequencing, and DNA–DNA hybridization studies (21). These species included *C. sakazakii, C. malonaticus, C. turicensis, C. muytjensii, C. dublinensis*, and *C*. genomospecies 1. A new species (*C. condimenti*) was subsequently identified and in addition *C. universalis* now replaces the original *C*. genomospecies 1 (31). Interestingly, it was proposed that three *Enterobacter* species, namely *E. pulveris*, *E. helveticus*, and *E. turicensis*, should be included in the genus *Cronobacter*, being designated as *C. pulveris*, *C. helveticus*, and *C. zurichensis*, respectively (32). This reclassification was based on a multi-locus sequence analysis (MLSA) scheme, using the concatenated nucleotide sequences of *gyrB*, *rpoB*, *infB*, and *atpD* genes to generate a phylogenetic tree, without any further phenotypic characterizations (32). All three species were originally isolated and characterized as *Enterobacter* species (33, 34) but excluded from the *Cronobacter* species classification due to their obvious phenotypic characteristics (35). More recently, the whole genome sequence (WGS) of these three isolates (36–38), along with a detailed re-examination of their taxonomic status, was reported (39). These data confirmed their exclusion from the genus *Cronobacter* and furthermore provided the evidence necessary to re-classify them as two new bacterial genera, designated as *Siccibater and Franconibacter*, respectively (39). Nonetheless, these studies demonstrate the considerable diversity with respect to both geno- and phenotypic characteristics among *Cronobacter* species and its close taxonomic neighbors, as well as the complexity and challenges, now confronting bacterial taxonomists.

**Figure 2:**
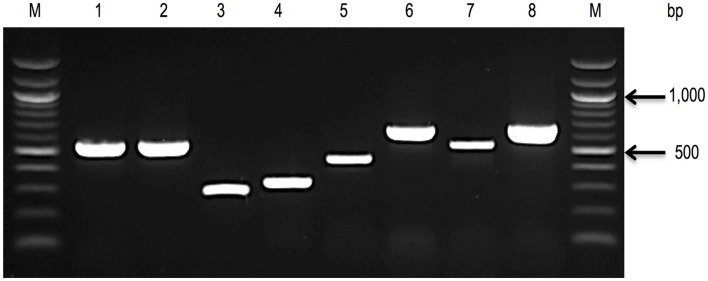
**A 1.0% agarose gel, showing *rpoB* amplicons, used to identify seven species of the genus (40, 41)**. Lane 1, *C. sakazakii* ATCC^®^ BAA-894; Lane 2, *C. malonaticus* E615 (PCR amplification using *rpoB C*. *sakazakii* primer pair); Lane 3, *C. malonaticus* E615 (a second PCR amplification using *rpoB* C. *malonaticus* primer pair); Lane 4, *C. muytjensii* ATCC^®^ 51329; Lane 5, *C. dublinensis* E187; Lane 6, *C. turicensis* E694; Lane 7, *C. universalis* E680; Lane 8, *C. condimenti* 1330; and Lane M, 100 bp DNA ladder.

PCR assays targeting species-specific SNPs associated with genes including *rpoB* (40, 41) and *cgcA* (42) have been developed to facilitate the detection of all seven recognized species within the *Cronobacter* genus. Figure 2 showed the applications of these molecular-based protocols for the identification of *Cronobacter* species using primers targeting the *rpoB* gene. Interestingly, using this *rpoB* protocol, *C. malonaticus* and *C. sakazakii* could not be differentiated and required a second PCR reaction to accurately identify each of these species. A multiplex PCR assay targeting the *cgcA* gene was developed to differentiate species within the genus *Cronobacter* and this protocol was found to be 100% specific and sensitive (42). Its advantage over the *rpoB* method is the ability to directly identity *C. sakazakii* and *C. malonaticus* in a single reaction. However, the recently described *C. condimenti* cannot be identified using the *cgcA* method (42).

Other biomarkers, in particular virulence genes, also have potential to be used as targets for species identification. Yan et al. (43) designed a PCR and array-based biomarker verification strategy for the detection and identification of *Cronobacter* species. This strategy was being proposed to facilitate the elucidation of virulence genes, which may be helpful as biomarkers for differentiating *Cronobacter* species from other food-borne pathogens. However, these putative markers are yet to be validated before their adoption.

### Serotype identification

The O-antigen is a component of the lipopolysaccharide (LPS) structure located on the outer surface of gram-negative bacteria, and is responsible for serological diversity. A molecular-based strategy to identify O-serotype associated with *Cronobacter* species was described previously (44–48). Mullane et al. (44) initially developed a molecular serotyping method, which included a long-range PCR amplification of the *rfb-*encoding locus (in Gram-negative enteric bacteria located between *galF* and *gnd* genes), followed by restriction fragment length polymorphism (RFLP) analysis using *Mbo*II. These digests were separated on a conventional agarose gel and visualized under UV light. A tiff image was generated and imported into BioNumerics (Applied Maths, Sint-Martens-Latem, Belgium). Using this approach, a PCR-RFLP profile of each isolate was produced, which can be compared across various isolates. The first two O-antigen molecular serotypes were denoted as O:1 and O:2 within *C. sakazakii* (44).

Later, another five additional O-antigens were identified in *C. sakazakii* by Sun et al. (46) and these correlated with the previously reported PCR-RFLP profiles. These molecular-characterization schemes were further extended to include other *Cronobacter* species and define new molecular O-serotype gene clusters (45, 48). To date, 15 *Cronobacter* serogroups were identified following the comparison of these PCR amplification schemes, which consist of *C. sakazakii* O:1–O:4 and O:7; *C. malonaticus* O:1–O:2; *C*. *dublinensis* O:1–O:2, *C. muytjensii* O:1–O:2, *C. universalis* O:1, as well as *C. turicensis* O:1–O:3 (Figure 1). Interestingly, some of the O-serotype gene clusters are shared among various species, such as *C. sakazakii* O:3 and *C. muytjensii* O:1, as well as *C. malonaticus* O:1 and *C. turicensis* O:1 (49).

## Sub-Typing Methods

Molecular sub-typing has long been regarded as a useful approach that can be applied to elucidate the nature of those bacteria colonizing a particular ecological niche. A number of strategies has been applied for the sub-tying of *Cronobacter* species, which include pulsed-field gel electrophoresis (PFGE), multi-locus sequence typing (MLST), multi-locus variable number tandem-repeat analysis (MLVA), multi-locus sequence analysis (MLSA), as well as matrix-assisted laser desorption ionization-time of flight mass spectrometry (MALDI-TOP MS) (50). PFGE, and MLST approaches are those most widely in use currently.

### Pulsed-field gel electrophoresis

**Figure 3:**
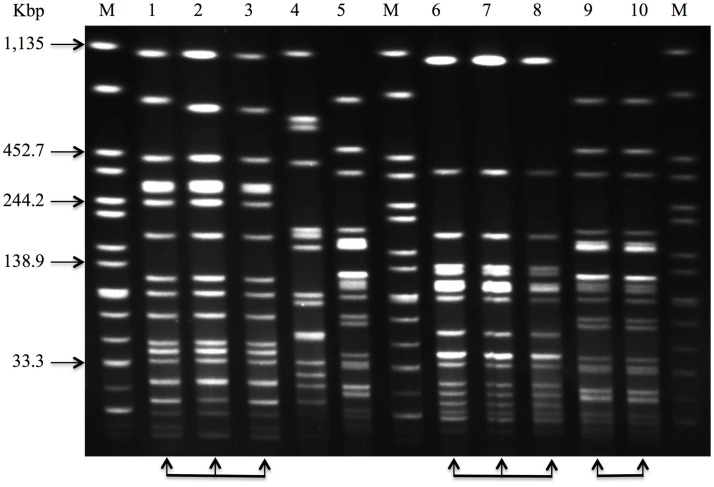
**Pulsed-field gel electrophoresis (PFGE) profiles that can be used to characterize and track *Cronobacter* species in a PIF processing facility**. Lane 1–10, sample 1–10, and Lane M, *Salmonella* Braenderup H9812, molecular marker, genomic DNA digested with *Xba*I. The arrow-heads at the foot of the image, including lane 1 through 3, lane 6 through 8, lane 9 and 10, show that these isolates have the same PFGE profile and would be considered indistinguishable.

Nazarowec-White and Farber (51) first applied PFGE to characterize and sub-type *Cronobacter* species. Mullane et al. (8) characterized and tracked *Cronobacter* species in a PIF processing facility using PFGE. The study provided a basis for the development of efficient intervention measures contributing to the reduction of *Cronobacter* in the PIF manufacturing environment. Since then, PFGE approaches have been widely used to track the movement of *Cronobacter* species in infant foods, soft cheese, various categories of ready-to-eat foods, farming and domestic environments, food producing animals, dried milk, and related products (4, 52–62). Meanwhile, a standardized PFGE protocol for sub-typing *Cronobacter* has been developed and validated by PulseNet, a network of national and regional laboratory sites dedicated to tracking food-borne infections (63). Figure 3 shows an agarose gel with *Cronobacter* isolates of diverse pulsotypes, cultured from such environments.

### Multi-locus sequence typing

**Figure 4:**
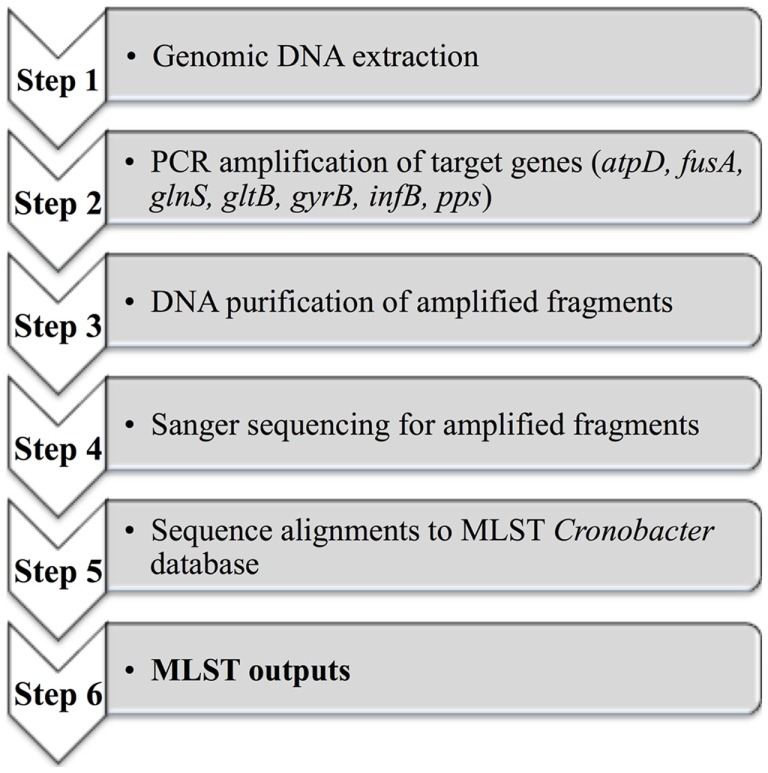
**A diagram outlining the MLST scheme from genomic DNA to the MLST outputs**.

A MLST scheme was originally developed by Baldwin et al. (64) to discriminate between *C. sakazakii* and *C. malonaticus*. This MLST strategy included seven housekeeping genes: *atpD, fusA, glnS, gltB, gyrB, infB, pps* (3,036 bp concatenated length) that could be used for phylogenetic analysis and comparative genomics (65). Joseph and Forsythe (66) reported the identification of a highly stable sequence type (denoted as ST4) within *C. sakazakii* and which was responsible for a large proportion of the documented severe neonatal infections, including neonatal meningitis. A database containing defined sequence types (ST) covering all *Cronobacter* species is currently maintained at the University of Oxford. This database and the associated MLST protocols can be accessed at www.pubMLST.org/cronobacter. Figure 4 described the application of MLST, which includes an initial genomic DNA extraction, PCR amplification of target genes, DNA purification of amplified fragments, Sanger sequencing, alignments to the MLST *Cronobacter* database, and finally MLST data outputs. This scheme has already been applied in epidemiologic investigations, screening for *Cronobacter* species in both commercial infant formula products and in hospital or industrial environments (60, 62, 67, 68). However, Pan et al. (62) reported that PFGE demonstrated a superior typing capability when compared with MLST and thereafter suggested a combined approach for the sub-typing of *Cronobacter* species from food and its related environments.

Additionally, combined with next generation sequencing (NGS), the *Cronobacter* PubMLST genome and sequence definition database^3^ was established, which covered over 1,000 isolates linked to neonatal meningitis and adult infections (69). These authors identified *C. sakazakii* clonal complex 4 (CC4) as principally associated with neonatal meningitis. This clonal lineage was confirmed using ribosomal-MLST and whole genome-MLST analysis.

## Sequencing the Genome of *Cronobacter* Species

Genome sequencing efforts can be expected to facilitate the correct identification of a bacterial species; it can also provide detailed information regarding the unique geno- and phenotypic features. Moreover, these approaches can be used for comparative purposes in order to rapidly and simultaneously investigate the presence/absence of all annotated genes or coding sequences (CDS), and/or nucleotide polymorphisms that may contribute to a specific morphology or physiology.

### Whole genome sequencing

Thirty-five *Cronobacter* genome sequences are currently available at the National Centre for Biotechnology Information (NCBI) as described in Table S1 in Supplementary Material. Only five *Cronobacter* genomes have been completed or closed, including the genomes of *C. sakazakii* ATCC^®^BAA-894, ES15, and SP291, *C. turicensis* z3032 (LMG 23827), as well as *C. malonaticus* CMCC 45402 (70–74).

*Cronobacter sakazakii* ATCC^®^BAA-894 was the first sequenced isolate (70), which was originally cultured from PIF and epidemiologically linked with an outbreak in a neonatal intensive care unit (75, 76). The genome sequence revealed a single chromosome of 4.4 Mbp along with two plasmids, denoted as pESA2 (31 kbp) and pESA3 (131 kbp). The isolate was compared with representatives of five other species using DNA microarray in an effort to further investigate the core genome of *Cronobacter* along with virulence factors. Among 4,392 annotated genes, some 43% of the genes were shared across five species, while 55% of the genes were unique to *C. sakazakii*. A copper and silver resistance system, which is known to be linked to invasion of the blood-brain barrier by neonatal meningitis causing strains of *E. coli* (77), was identified in isolates associated with neonatal infections (including *C. sakazakii, C. malonaticus*, and *C. turicensis*). In particular, genes encoding multidrug efflux pumps and adhesins were found to be unique to *C. sakazakii* ATCC^®^BAA-894 (70).

More recently, the complete genome of *C. sakazakii* SP291 was reported (73, 78). This isolate was cultured from a PIF production environment and it represented a cluster of isolates, which were found to be persistent in the PIF production site for at least a period of 2 years. The genome of *C. sakazakii* SP291 included a 4.3 Mbp chromosome and three plasmids, denoted as pSP291 (118 kbp), pSP291-2 (52 kbp), and pSP291-3 (4 kbp). Compared with *C. sakazakii* ATCC^®^BAA-894, *C. sakazakii* SP291 exhibited a markedly better series of stress response mechanisms (73). Given the fact that *C. sakazakii* SP291 adapted to the stressful PIF production environment, the osmoprotectant ABC transporters, including YehZYXW, ProP, ProU, and OpuCABCD, can be expected to play an important role in supporting its survival (73), a feature that has been functionally confirmed in other microorganisms (79–82). Furthermore, a greater ability to survive in a broader range of heavy metals was also noted for *C. sakazakii* SP291, which may be accounted for by its frequent exposure to quaternary ammonium-containing disinfectants (73).

*Cronobacter sakazakii* ES15 was cultured from ground whole grains, and subsequently sequenced (72). In this case the genome consisted of a single chromosome of 4.3 Mbp devoid of any plasmids. Interestingly, a relatively high number of ABC transport systems and phosphotransferase systems (PTS) were identified in this genome, which may possibly suggest the existence of efficient nutrient uptake systems. In additional, OmpA reported to be involved in the basolateral invasion of the brain by *C. sakazakii* (83) was also identified.

This isolate *C. turicensis* z3032, linked with the deaths of two newborn infants in Switzerland in 2005, was subsequently cultured from the blood of one child with meningitis. The genome of this isolate was investigated in an effort to further determine those virulence factors and mechanisms associated with the pathogenicity of these isolates (71). The genome was found to be 4.4 Mbp in size and contained three plasmids of sizes 138 kbp (pCTU1), 22 kbp (pCTU2), and 54 kbp (pCTU3). In all, 4,455 CDS were annotated, with 5% of them being virulence- and disease-related (71).

The latest WGS to be published was that of *C. sakazakii* CMCC 45402 (74), which was believed to be *C. malonaticus* based on the neighbor-joint likelihood phylogeny (13). The *rpoB* gene of *C. malonaticus* matched the CMCC 45402 draft genome completely, and which suggested that it was originally mis-identified and is in fact a *C. malonaticus* isolate. This genome included a 4.4 Mbp chromosome with two plasmids of 127 kbp (denoted as p1) and 56 kbp (denoted as p2) in length. The isolate was cultured from a milk sample in China. Genes involved in pathways, such as microbial metabolism in diverse environments, purine metabolism, and ABC transporter pathways were identified (74).

Grim et al. (84) reported on a comparative genomic analysis of six species of *Cronobacter* in an attempt to understand the evolution of these bacteria and the genetic contents of each species. A total of 3,160 CDS comprised the core genome of the *Cronobacter* species (84), which was considerably more than the original 1,899 genes identified using DNA microarray across five species (70). Eighty-four dispensable genomic regions (defined as containing genes present in two or more strains) were also determined. According to Medini et al. (85), the pan genome consists of the sum of the core genome, which includes all genes responsible for the basic aspects of the biology of a species and its major phenotypic traits, as well as dispensable genomes, which contributes to the species diversity and may encode supplementary biochemical pathways and functions that are not essential for bacterial growth but confer selective advantages, such as adaption to different niches, antibiotic resistance, or colonization of a new host. Most notably, several type VI secretion system gene clusters, transposons that carried tellurium, copper and/or silver resistance genes, along with a novel integrative conjugative element (ICE), were identified (84). Furthermore, *Cronobacter* appeared to have diverged into two clusters, one consisting of *C. dublinensis* and *C. muytjensii* (Cdub-Cmuy) and the other comprised of *C. sakazakii*, *C. malonaticus*, *C. universalis*, and *C. turicensis* (Csak-Cmal-Cuni-Ctur). The Cdub-Cmuy clade contained several accessory genomic regions important for survival in a plant-associated environmental niche, while the Csak-Cmal-Cuni-Ctur clade genomes harbored numerous virulence-related genetic traits (84).

### Plasmid sequencing

Numerous plasmids have been identified in the five completed genomes of *Cronobacter*, including pESA2 and pESA3 in *C. sakazakii* ATCC^®^BAA-894, pSP291-1, pSP291-2, and pSP291-3 in *C. sakazakii* SP291, pCTU1, pCTU2, and pCTU3 in *C. turicensis* z3032, as well as p1 and p2 in *C. malonaticus* CMCC 45402. Yan et al. (73) classified these plasmids into two groups initially, based on their similarity following alignments. With further analysis performed more recently to include those new plasmids that were reported, *plasmid group 1* now contains of plasmids pESA3 from *C. sakazakii* ATCC^®^BAA-894, pSP291-1 from *C. sakazakii* SP291, p1 from *C. malonaticus* CMCC 45402, and pCTU1 from *C. turicensis* z3032. This group carries two arsenical resistance genes and several putative virulence genes, including two genetic loci encoding iron acquisition systems, namely an ABC transporter gene cluster and an aerobactin or cronobactin siderophore receptor gene cluster identified as *eitCBAD* and *iucABCD*/*iutA*, respectively (73). The *iucABCD/iutA* is reported to be the only active siderophore present in *Cronobacter* (84, 86). Additionally *plasmid group 2* now includes plasmids pSP291-2 from *C. sakazakii* SP291, p2 from *C. malonaticus* CMCC 45402, and pCTU3 from *C. turicensis* z3032. Fifteen heavy metal (copper, cobalt, zinc, cadmium, lead, and mercury) resistance genes, an osmosensitive K^+^ channel histidine kinase gene *kdpD* and a virulence associated gene *vagC* were broadly shared among these plasmids (73). Furthermore, the presence of a *Cronobacter* plasminogen activator-encoding gene (*cpa*) [encoded on plasmids pESA3 and pSP291-1], a single RepFIB-like origin of replication gene (*repA*) [encoded on pESA3 and pCTU1], a type VI secretion system (T6SS) [encoded on pESA3], a filamentous hemagglutinin/adhesin (FHA) gene locus (located on pCTU1), membrane proteins, suppressor of copper-sensitivity (*scsA* and *scsB*) [shared among pESA3, p1, and pSP291-1], seven arsenical resistance genes (shared between p2 and pCTU3) suggested the existence of unique virulence determinants in these species (73). Other plasmids, including pESA2, pSP291-3, and pCTU2 demonstrated no similarity to any of the above groups, as determined following their alignments and analysis (73).

### Microarray-based technologies and deep-level RNA sequencing

DNA Microarray has been applied to investigate the genetic diversity of *Cronobacter* species previously (70, 87). Healy et al. (87) initially designed and performed a microarray-based analysis of *Cronobacter* species using 276 open reading frame selected from *C. sakazakii* ATCC^®^BAA-894 to identify species-specific genes that could be evaluated as candidate markers for inclusion in a molecular-based detection protocol. After completing the WGS of *C. sakazakii* ATCC^®^BAA-894, Kucerova et al. (70) constructed a 387,000 probe oligonucleotide microarray in an effort to identify the pan-genome of *Cronobacter* using five of the seven recognized species. More recently, US-FDA developed a custom designed multi-genome DNA microarray platform that contains over 21,402 unique genes (470,844 probes), representing the pan genome of all seven *Cronobacter* species (88). Early results showed its capacity to distinguish all seven *Cronobacter* species from one another and from closely related non-*Cronobacter* species (88).

High-throughput whole-transcriptome sequencing (RNA-seq) has also been performed to characterize and fingerprint *C. sakazakii* responses following exposure to two garlic-derived organosulfur compounds, ajoene and diallyl sulfide (89). Interestingly, RNA-seq revealed that bacteria response to the two compounds differ. For example, ajoene caused downregulation of motility-related genes, while diallyl sulfide treatment caused an increased expression of cell wall synthesis genes. These findings will aid the development of effective intervention strategies to decrease the risk of *Cronobacter* contamination in the food production environments and contact surfaces.

## Future Directions

*Cronobacter* species, like other microorganisms, can adapt to the production environment. Previous studies reported the isolation of *Cronobacter* from PIF and its production environment, suggesting that this bacterium has the capacity to adapt to, survive, and persist under desiccated environmental conditions (53, 90). With the advantages afforded by NGS technology, isolates of interest can now be investigated in considerable detail. Additionally, the stress response factors identified previously in *Cronobacter* species, such as heat-shock, cold-stresses, survival in dry conditions, water activity (a_w_), and pH need to be re-assessed using RNA-seq and other novel approaches that are currently under development. Advances in our understanding of mechanisms involved with *Cronobacter* survival will be the key to developing better food safety measures to reduce the risk of *Cronobacter* contamination in PIF and its production environments and to protect neonatal health.

## Conflict of Interest Statement

The authors declare that the research was conducted in the absence of any commercial or financial relationships that could be construed as a potential conflict of interest.

## Supplementary Material

The Supplementary Material for this article can be found online at http://journal.frontiersin.org/article/10.3389/fped.2015.00038

## Footnotes

^1^http://www.iso.org/iso/catalogue_detail.htm?csnumber=41258

^2^http://www.fda.gov/Food/FoodScienceResearch/LaboratoryMethods/ucm289378.htm

^3^http://www.pubMLST.org/cronobacter
