# Supplementary material for: Strategies for the Identification and Tracking of Cronobacter Species: An Opportunistic Pathogen of Concern to Neonatal Health
Source: Front Pediatr. 2015 May 5;3:38. doi: 10.3389/fped.2015.00038 (PMC4419663; doi:10.3389/fped.2015.00038)
Supplement: Supplementary file 1 [file Table_1.DOCX]

**Supplementary Table S1** Genome sequencing projects reported for *Cronobacter* species available at NCBI–March 24, 2015

| **Strain ID** | **Size (Mb)** | **GC(%)** | **NCBI Reference** | **Plasmid** | **Scaffold** | **Gene** | **Protein** | **Release Date** | **Reference** |
| --- | --- | --- | --- | --- | --- | --- | --- | --- | --- |
| *C. sakazakii* ATCC^®^ BAA-894 | 4.53 | 56.8 | NC_009778.1; NC_009780.1; NC_009779.1 | pESA2; pESA3 | 3 | 4,562 | 4,422 | 28/08/2007 | [Kucerova et al. (2010](#_ENREF_5)) |
| *C. sakazakii* ES15 | 4.27 | 57.1 | NC_017933.1 | - | 1 | 4,018 | 3,916 | 17/05/2012 | [Shin et al. (2012](#_ENREF_8)) |
| *C. sakazakii* SP291 | 4.52 | 56.8 | NC_020260.1; NC_020262.1; NC_020263.1; NC_020261.1 | pSP291-1; pSP291-2; pSP291-3 | 4 | 4,286 | 4,182 | 11/02/2013 | [Yan et al. (2013](#_ENREF_11)) |
| *C. sakazakii* E899 | 3.96 | 57.5 | AFMO01 | - | 385 | 3,718 | 3,629 | 25/05/2011 | [Chen et al. (2011](#_ENREF_1)) |
| *C. sakazakii* E696 | 4.99 | 55.9 | CALF01 | - | 569 | 4,724 | 4,659 | 15/11/2012 | [Joseph et al. (2012](#_ENREF_4)) |
| *C. sakazakii* E701 | 4.85 | 55.8 | CALE01 | - | 768 | 4,540 | 4,471 | 15/11/2012 | [Joseph et al. (2012](#_ENREF_4)) |
| *C. sakazakii* E680 | 4.36 | 56.8 | CALG01 | - | 201 | 4,218 | 4,143 | 15/11/2012 | [Joseph et al. (2012](#_ENREF_4)) |
| *C. sakazakii* E764 | 4.44 | 57.0 | AJLA01 | - | 32 | - | - | 07/06/2013 | [Grim et al. (2013](#_ENREF_2)) |
| *C. sakazakii* 2151 | 4.38 | 57.0 | AJKT01 | - | 60 | - | - | 07/06/2013 | [Grim et al. (2013](#_ENREF_2)) |
| *C. sakazakii* ES713 | 4.55 | 56.7 | AJLB01 | - | 156 | - | - | 07/06/2013 | [Grim et al. (2013](#_ENREF_2)) |
| *C. sakazakii* ES35 | 4.35 | 57.0 | AJLC01 | - | 183 | - | - | 07/06/2013 | [Grim et al. (2013](#_ENREF_2)) |
| *C. sakazakii* NCIMB 8272 | 4.58 | 56.8 | AWFW01 | - | 82 | - | - | 30/08/2013 | [Masood et al. (2013b](#_ENREF_7)) |
| *C. sakazakii* 8399 | 4.66 | 56.6 | AWSP01 | - | 33 | - | - | 16/09/2013 | [Masood et al. (2013a](#_ENREF_6)) |
| *C. sakazakii* NBRC 102416 | 4.55 | 56.7 | BAWU01 | - | 100 | - | - | 24/04/2014 | [Hosoyama et al. (2014](#_ENREF_3)) |
| *C. sakazakii* HPB5174 | 4.44 | 56.9 | JNBN01 | - | 84 | 4,136 | 3,993 | 04/06/2014 | [Tatusova et al. (2014](#_ENREF_10)) |
| *C. dublinensis* 1210 | 4.6 | 57.8 | CAKZ01 | - | 221 | 4,423 | 4,342 | 15/11/2012 | [Joseph et al. (2012](#_ENREF_4)) |
| *C. dublinensis* 582 | 4.76 | 57.3 | CALA01 | - | 427 | 4,516 | 4,444 | 15/11/2012 | [Joseph et al. (2012](#_ENREF_4)) |
| *C. dublinensis* LMG 23823 | 4.58 | 57.8 | AJKZ01 | - | 82 | - | - | 07/06/2013 | [Grim et al. (2013](#_ENREF_2)) |
| *C. dublinensis* LMG 23825 | 4.45 | 58.3 | AJKX01 | - | 95 | - | - | 07/06/2013 | [Grim et al. (2013](#_ENREF_2)) |
| *C. dublinensis* LMG 23824 | 4.61 | 57.9 | AJKY01 | - | 105 | - | - | 07/06/2013 | [Grim et al. (2013](#_ENREF_2)) |
| *C. turicensis* z3032 | 4.6 | 57.2 | NC_013282.2; NC_013283.1; NC_013284.1; NC_013285.1 | pCTU1; pCTU2; pCTU3 | 4 | 4,562 | 4,452 | 02/10/2009 | [Stephan et al. (2011](#_ENREF_9)) |
| *C. turicensis* 564 | 4.57 | 57.2 | CALB01 | - | 114 | 4,316 | 4,244 | 15/11/2012 | [Joseph et al. (2012](#_ENREF_4)) |
| *C. malonaticus* CMCC 45402 | 4.56 | 56.8 | NC_023032.1; NC_023025.1; NC_023024.1 | p1; p2 | 3 | 4,417 | 4,313 | 09/12/2013 | [Zhao et al. (2014](#_ENREF_12)) |
| *C. malonaticus* 681 | 4.55 | 56.5 | CALC01 | - | 171 | 4,325 | 4,254 | 15/11/2012 | [Joseph et al. (2012](#_ENREF_4)) |
| *C. malonaticus* 507 | 4.53 | 56.6 | CALD01 | - | 249 | 4,246 | 4,192 | 15/11/2012 | [Joseph et al. (2012](#_ENREF_4)) |
| *C. malonaticus* LMG 23826 | 4.43 | 56.8 | AJKV01 | - | 127 | - | - | 07/06/2013 | [Grim et al. (2013](#_ENREF_2)) |
| *C. malonaticus* 685 | 4.60 | 56.8 | JPZD01 | - | 352 | 4,456 | 4,313 | 20/08/2014 | [Tatusova et al. (2014](#_ENREF_10)) |
| *C. malonaticus* 687 | 4.41 | 56.9 | JPZE01 | - | 35 | 4,061 | 3,943 | 20/08/2014 | [Tatusova et al. (2014](#_ENREF_10)) |
| *C. malonaticus* 1545 | 4.55 | 56.7 | JPZF01 | - | 470 | 4,425 | 4,286 | 20/08/2014 | [Tatusova et al. (2014](#_ENREF_10)) |
| *C. malonaticus* 1558 | 4.47 | 56.9 | JPZG01 | - | 309 | 4,275 | 4,135 | 20/08/2014 | [Tatusova et al. (2014](#_ENREF_10)) |
| *C. malonaticus* 1569 | 4.53 | 57.0 | JPZH01 | - | 466 | 4,368 | 4,199 | 20/08/2014 | [Tatusova et al. (2014](#_ENREF_10)) |
| *C. malonaticus* ENBT0334 | 4.47 | 57.0 | JXTD01 | - | 128 | 4,160 | 3,904 | 20/02/2015 | Timme et al. (2015) |
| *C. condimenti* 1330 | 4.48 | 55.8 | CAKW01 | - | 155 | 4,350 | 4,272 | 03/12/2012 | [Joseph et al. (2012](#_ENREF_4)) |
| *C. muytjensii* ATCC^®^ 51329 | 4.32 | 57.7 | AJKU01 | - | 72 | - | - | 17/06/2013 | [Grim et al. (2013](#_ENREF_2)) |
| *C. universalis* NCTC 9529 | 4.52 | 57.4 | CAKX01 &AJKW01 | - | 231 | 4,357 | 4,285 | 15/11/2012 | [Grim et al. (2013](#_ENREF_2)); [Joseph et al. (2012](#_ENREF_4)) |

**Reference**

Chen, Y., Strain, E.A., Allard, M., and Brown, E.W. (2011). Genome sequence of *Cronobacter sakazakii* E899, a strain associated with human illness. *J Bacteriol* 193**,** 5861. doi: 10.1128/JB.05913-11.

Grim, C.J., Kotewicz, M.L., Power, K., Pagotto, F., Gopinath, G., Mammel, M.K., Jarvis, K.G., Yan, Q.Q., Kothary, M.H., Franco, A.A., Patel, I.R., Jackson, S.A., Hu, L., Sathyamoorthy, V., Iversen, C., Lehner, A., Stephan, R., Farber, J.M., Fanning, S., and Tall, B.D. (2013). Pan genome analysis of the emerging foodborne pathogen *Cronobacter* spp. suggests a species-level bidirectional divergence driven by niche adaption. *BMC Genomics* 14**,** 366.

Hosoyama, A., Hashimoto, M., Hosoyama, Y., Noguchi, M., Numata, M., Tsuchikane, K., Hirakata, S., Uohara, A., Ohji, S., Ichikawa, N., Yamazoe, A., and Fujita, N. (2014). Whole genome shotgun sequence of *Cronobacter sakazakii* NBRC 102416. *Unpublished*.

Joseph, S., Desai, P., Ji, Y., Cummings, C.A., Shih, R., Degoricija, L., Rico, A., Brzoska, P., Hamby, S.E., Masood, N., Hariri, S., Sonbol, H., Chuzhanova, N., Mcclelland, M., Furtado, M.R., and Forsythe, S.J. (2012). Comparative analysis of genome sequences covering the seven *Cronobacter* species. *PloS One* 7**,** e49455. doi: 10.1371/journal.pone.0049455.

Kucerova, E., Clifton, S.W., Xia, X.Q., Long, F., Porwollik, S., Fulton, L., Fronick, C., Minx, P., Kyung, K., Warren, W., Fulton, R., Feng, D.Y., Wollam, A., Shah, N., Bhonagiri, V., Nash, W.E., Pepin, K.H., Wilson, R.K., Mcclelland, M., and Forsythe, S.J. (2010). Genome sequence of *Cronobacter sakazakii* BAA-894 and comparative genomic hybridization analysis with other *Cronobacter* species. *Plos One* 5**,** 1-10. doi: 10.1371/journal.pone.0009556.t001.

Masood, N., Moore, K., Farbos, A., Hariri, S., Block, C., Paszkiewicz, K., Dickins, B., Mcnally, A., and Forsythe, S. (2013a). Draft genome sequence of a meningitic isolate of *Cronobacter sakazakii* clonal complex 4, strain 8399. *Genome Announc* 1**,** e00833-00813.

Masood, N., Moore, K., Farbos, A., Hariri, S., Paszkiewicz, K., Dickins, B., Mcnally, A., and Forsythe, S. (2013b). Draft genome sequence of the earliest *Cronobacter sakazakii* sequence type 4 Strain, NCIMB 8272. *Genome Announc* 1**,** e00782-00713.

Shin, H., Lee, J.H., Choi, Y., and Ryu, S. (2012). Complete genome sequence of the opportunistic food-borne pathogen *Cronobacter sakazakii* ES15. *J Bacteriol* 194**,** 4438-4439. doi: 10.1128/JB.00841-12.

Stephan, R., Lehner, A., Tischler, P., and Rattei, T. (2011). Complete genome sequence of *Cronobacter turicensis* LMG 23827, a food-borne pathogen causing deaths in neonates. *J Bacteriol* 193**,** 309-310. doi: 10.1128/JB.01162-10.

Tatusova, T., Ciufo, S., Fedorov, B., O'neill, K., and Tolstoy, I. (2014). RefSeq microbial genomes database: new representation and annotation strategy. *Nucleic Acids Res* 42**,** D553-559.

Timme, R., Allard, M.W., Strain, E., Evans, P.S., and Brown, E. (2015). Whole genome shotgun sequencing of cultured foodborne pathogen. http://www.ncbi.nlm.nih.gov/nuccore/758923693.

Yan, Q.Q., Power, K.A., Cooney, S., Fox, E., Gopinath, G.R., Grim, C.J., Tall, B.D., Mccusker, M.P., and Fanning, S. (2013). Complete genome sequence and phenotype microarray analysis of *Cronobacter sakazakii* SP291: a persistent isolate cultured from a powdered infant formula production facility. *Front Microbiol* 4**,** 256. doi: 10.3389/fmicb.2013.00256.

Zhao, Z.J., Wang, L., Wang, B., Liang, H.Y., Ye, Q., and Zeng, M. (2014). Complete genome sequence of *Cronobacter sakazakii* strain CMCC 45402. *Genome Announc* 2**,** e01139-01113 doi: 10.1128/genomeA.01139-13.
